# Can a 3 months treatment with oral Desogestrel prior to insertion of the etonogestrel-releasing contraceptive implant improve continuation rate at 1 year? A randomized trial

**DOI:** 10.1186/s13104-023-06304-3

**Published:** 2023-03-13

**Authors:** Valeria Lombardi Fäh, Rosa Catarino, Sarah Castillo, Maria Badda, Sibel Gezer-Dickschat, Friederike Thieringer, Sibil Tschudin, Manuela Viviano, Michal Yaron

**Affiliations:** 1 Service of Gynecology, Department of Woman, Child and Adolescent, Geneva University Hospitals, Boulevard de La Cluse 30, 1205 Geneva, Switzerland; 2 Service of Gynecology and Obstetrics, Hospital Neuchâtel–Pourtalès, Rue de La Maladière 45, 2000 Neuchâtel, Switzerland; 3 Service of Gynecology and Obstetrics, GHOL - Hospital Nyon, Chemin Monastier 10, 1260 Nyon, Switzerland; 4 Service of Gynecology and Obstetrics, University Hospital Basel, Universitätsspital CH, Petersgraben 4, 4031 Basel, Switzerland

**Keywords:** Desogestrel pill, Discontinuation, Long-acting reversible contraceptives (LARC), Tolerance, Etonogestrel-releasing contraceptive implant

## Abstract

**Objective:**

To evaluate if daily oral 75 µg of Desogestrel (DSG) for 3 months prior to the insertion of etonogestrel-releasing contraceptive implant (ENG-IMPLANT) might help reduce its premature discontinuation.

**Results:**

A total of 66 women were randomized in the ENG-IMPLANT group (26) and in the DSG + ENG-IMPLANT group (40), respectively, in the Geneva University Hospitals and Basel University Hospital, from August 15th, 2016 through September 30th, 2019. In the DSG + ENG-IMPLANT group, patients were given a 3 months’ supply of 75 µg of DSG before the insertion of the ENG-IMPLANT. All women were seen after 3 months for bleeding and satisfaction evaluation, and at 12 months post ENG-IMPLANT insertion. Higher levels of satisfaction at 12-months were found in the ENG-IMPLANT group compared to the DSG + ENG-IMPLANT group (8.5 ± 1.7 vs. 6.6 ± 2.9, p = 0.012). There were no statistically significant differences regarding tolerance (7.8 ± 2.5 vs 6.8 ± 2.6, p = 0.191) and contraceptive continuation (80% vs 72.4%, p = 0.544) between groups.

**Conclusion:**

DSG prior to insertion of the ENG-IMPLANT did not improve its continuation rate neither its satisfaction at 1 year.

*Trial registration* NCT05174195. Retrospectively registered, the 30th December 2021

**Supplementary Information:**

The online version contains supplementary material available at 10.1186/s13104-023-06304-3.

## Introduction

Etonogestrel-releasing contraceptive implant (ENG-IMPLANT) is a single rod implant containing 68 mg of etonogestrel which is user independent and can be used for three years. Main side effects of ENG-IMPLANT are related to irregular vaginal bleeding. Other side effects such as weight gain, headache, acne, mastodynia, abdominal pain, emotional liability and vaginal infection are less frequent [1]. These side effects reduce tolerance of implants, which can lead to its premature removal [2]. The results of 11 international studies showed a continuation rate of 65% at 12 months [3], which is similar to data described in other European [4] and worldwide studies [5, 6].

An approach that would triage women who may have side effects and consequently difficulties adhering to ENG-IMPLANT could decrease premature removal. Such approach could be the use of daily oral 75 µg of Desogestrel (DSG) prior to the insertion of ENG-IMPLANT. In fact, DSG is transformed into its active form, etonogestrel, after hepatic metabolism and a very similar side effect profile has been described between ENG-IMPLANT and DSG users [7]. An initial treatment with DSG prior to ENG-IMPLANT insertion may indicate the presence of individual intolerance. In other words, women who will not tolerate the side effects induced by DSG will be probably less inclined to have an ENG-IMPLANT insertion and opt for another contraceptive. With this approach a more tolerant population is believed to be attained, leading to less premature removal of the more expensive ENG-IMPLANT, supporting a better cost-effective choice.

This prospective randomized study aims to evaluate if the pre-treatment with DSG prior to ENG-IMPLANT insertion (I) increases the continuation rate at one year, if it (II) increases the tolerance of the implant and (III) to compare side effects profile of DSG and ENG-IMPLANT at 3 months.

## Main text

### Materials and methods

#### Setting and study population

Between August 15th, 2016 and September 30th, 2019, we conducted a randomized prospective open-label study of women aged 18 to 42 years who were interested in using ENG-IMPLANT and were willing to have 90 days of pre-treatment with DSG. The study was conducted in the family planning clinic of the Geneva University Hospitals and women’s policlinic of the University Hospital Basel. The exclusion criteria were pregnancy, lactation, vaginal bleeding of unknown origin, wish to become pregnant, weight > 80 kg, history of venous thromboembolism, hypertension, diabetes or other metabolic diseases, coagulation disorders, severe hepatic disorder, history of gynaecological cancer, known hypersensitivity to study drugs and current treatment with enzyme inducing drugs. The study was approved by the ethics committees of both hospitals (CCER 16-972). All patients signed an informed consent prior to recruitment.

#### Study procedure

Once consent was obtained, women were randomized either to the ENG-IMPLANT group or the DSG + ENG-IMPLANT group. In the ENG-IMPLANT group, the implant was inserted immediately. In the DSG + ENG-IMPLANT group, patients were given a 3 months’ supply of DSG to be started immediately after which insertion of ENG-IMPLANT was proposed. In both groups, patients were instructed to complete a bleeding calendar and a satisfaction questionnaire. A 3-month visit was pre-programmed for all participants, during which the bleeding calendar and the questionnaire were collected. Participants in the DSG + ENG-IMPLANT group who completed the 3 months on DSG pill had their ENG-IMPLANT inserted during this visit. All patients were seen or called over the phone after 12 months post ENG-IMPLANT insertion in both groups. Women who had their implant removed between 3 to 12 months after the insertion completed the satisfaction questionnaire at the time of removal.

#### Measurement of outcomes and other variables of interest

Socio-demographic characteristics were collected. Data collectors recorded the date of implant insertion or the date of DSG initiation. The main outcome variable for this study was method discontinuation. Secondary outcomes were the side effects, tolerance and satisfaction 3 months after inclusion and 12 months after ENG-IMPLANT insertion.

A satisfaction questionnaire and a bleeding calendar was used to assess bleeding patterns throughout the first 3 months of use in both groups. Overall, irregular vaginal bleeding was defined as any deviation from their habitual menstrual patterns of bleeding.

The satisfaction questionnaire evaluated side effects on skin, mood, sex-drive, abdomino-pelvic or breast pain, headaches, changes to vaginal discharge, and irregular vaginal bleeding according to a graded 5 points Likert score, from never experiencing (1 point) to all the time experiencing (5 points). Two separate questions evaluated tolerance and satisfaction and were graded on a scale from 0 to 10 (0 not tolerating or unsatisfied with the method and 10 being totally satisfied and perfectly tolerating). Weight gain as cause for removal was noted when relevant.

#### Randomization and statistical analysis

Statistical analysis was performed using the Stata program version 13 (StataCorp LP: College Station, TX, USA); the significance level for all tests was p < 0.05. To detect a mean difference of 0.5 standard deviation on the numerical tolerance scale, with a power of 0.85 and type 1 error rate of 0.05, we needed 2 groups of 74 participants. The randomization plan was so as to have 33% more participants in the DSG + ENG-IMPLANT group, with a 1:3 ratio, because of 35% drop out in the ENG-IMPLANT group. This would have provided a power of 0.8 to detect a difference in proportions with implant removal of 0.35 versus 0.15 to compensate for possible loss to follow-up. Sample size was set to 80 participants in the ENG-IMPLANT only group, and 120 in the DSG + ENG-IMPLANT group. Randomization was conducted on www.randomization.com using randomly permuted blocs of 14, 21 and 28 patients. The participants' study allocation was included in opaque, sealed envelopes, prepared by the Clinical Research Platform (PGO) in the University Hospital of Geneva.

For continuous variables, means and standard deviation (SD) were calculated; for categorical data, proportions were calculated. The chi-square test and Fisher´s exact test were used when appropriate, as well as the student’s *t*-test and Mann–Whitney test. Descriptive statistics were used to analyse the baseline characteristics of the study population.

### Results

#### Study population

**Fig. 1:**
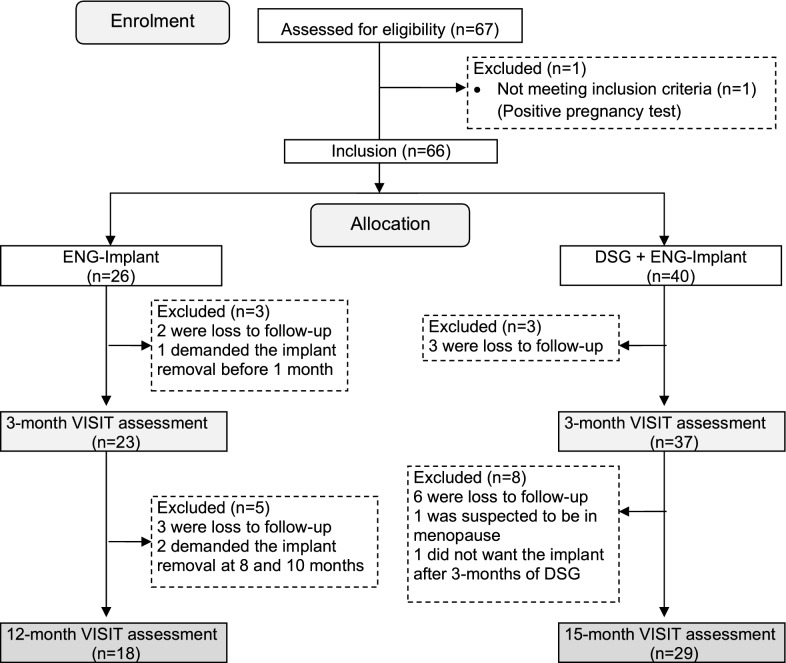
Flowchart of study participants

Among the 67 women assessed for eligibility, 66 (98.5%) were included in the study and were allocated either to the ENG-IMPLANT group (26, 39.4%) or the DSG + ENG-IMPLANT group (40, 60.6%) (Fig. 1). After 3 years of recruitment, 33.5% of the expected patients were recruited (67 patients instead of 200 patients as calculated in the sample size), Patient recruitment was more difficult than expected and after 3 years decision was made to terminate the study. Loss to follow-up at 3 months was similar in the two groups: 2 missed patients in the ENG-IMPLANT group and 3 in the DSG + ENG-IMPLANT group. Loss to follow-up at 12 months was also similar between groups: 3 additional missed patients in the ENG-IMPLANT group and 6 in the DSG + ENG-IMPLANT group. Overall, 1 patient was excluded before the 3-months assessment in the ENG-IMPLANT group because removal of the implant in less than a month after insertion (wish to conceive) and 2 patients did not complete the second questionnaire after demanding premature implant removal.

#### Participants' characteristics

Additional file 1: Table S1 summarizes participants’ characteristics in each group (total N = 60). The median age was 25 years (IQR: 20–31). Most of the women were nulliparous (73.3%). There were no statistical differences in the distribution of sociodemographic characteristics between the two groups.

#### Patients’ satisfaction and contraceptive side effects at 3 and 12- months’ assessment

Questionnaires about satisfaction were filled by 60 patients at 3-months and by 47 patients at 12-months (Table 1). There was no statistically significant difference between the two groups with respect to side effects. Women in the ENG-IMPLANT group reported a higher level of satisfaction at 12-months than the DSG + ENG-IMPLANT group (8.5 ± 1.7 vs. 6.6 ± 2.9, p = 0.012). There was no statistically significant difference regarding average tolerance between groups. Overall, the desire to continue the contraceptive method at 12 months was reported by 80.0% of women in the ENG-IMPLANT and 72.4% of women in the DSG + ENG-IMPLANT group (p = 0.544). At 12 months, 9 women in total (23.0%) did not want to continue with the ENG-IMPLANT. The reasons evoked for discontinuation were irregular vaginal bleeding (27.3%), weight gain (9.1%), multiple side effects (54.6%) and desire for pregnancy (9.1%). There were no differences in motives for discontinuation between the two groups.

**Table 1:** Satisfaction questionnaires results at 3 and 12- months assessment among the two groups (n = 60 at 3 months, n = 47 at 12 months)

Variable	3 M		P value	12 M		P value
	ENG-Implant	DSG + ENG-Implant		ENG-Implant	DSG + ENG-Implant	
Total	23	37		18	29	
Weight gain			0.181			0.571
No	13 (59.1)	28 (75.7)		10 (58.8)	13 (50.0)	
Yes	9 (40.9)	9 (24.3)		7 (41.2)	13 (50.0)	
Weight gain (kg), mean ± sd	1.1 ± 1.2	0.8 ± 1.4	0.209	3.1 ± 3.4	3.5 ± 3.1	0.704
Headache			0.724			0.711
Never or rarely	15 (65.2)	24 (64.8)		15 (83.3)	22 (78.6)	
Often to frequently	8 (34.8)	12 (32.4)		3 (16.7)	5 (17.8)	
All the time	0	1 (2.7)		0	1 (3.6)	
Acne			0.538			0.516
Never or rarely	12 (54.5)	23 (62.2)		15 (83.3)	22 (75.9)	
Often to frequently	7 (31.8)	12 (32.4)		3 (16.7)	5 (17.2)	
All the time	3 (13.7)	2 (5.4)		0	2 (6.9)	
Abdominal pain/bloating			0.378			0.543
Never or rarely	12 (52.2)	22 (59.5)		15 (83.3)	22 (75.9)	
Often to frequently	11 (47.8)	13 (35.1)		3 (16.7)	7 (24.1)	
All the time	0	2 (5.4)		0	0	
Mastodynia			0.191			0.180
Never or rarely	21 (91.3)	29 (78.4)		16 (88.9)	21 (72.4)	
Often to frequently	2 (8.7)	8 (21.6)		2 (11.1)	8 (27.6)	
All the time	0	0		0	0	
Vaginal infection			0.793			0.843
Never or rarely	20 (87.0)	33 (89.2)		15 (83.3)	25 (89.3)	
Often to frequently	3 (13.0)	4 (10.8)		2 (11.1)	2 (7.1)	
All the time	0	0		1 (5.6)	1 (3.6)	
Mood Swings			0.552			0.253
Never or rarely	10 (43.5)	21 (56.8)		12 (66.7)	16 (55.2)	
Often to frequently	12 (52.2)	14 (37.8)		6 (33.3)	9 (31.0)	
All the time	1 (4.3)	2 (5.4)		0	4 (13.8)	
Reduced sex drive			0.718			0.842
Never or rarely	14 (60.9)	25 (69.4)		11 (61.1)	20 (69.0)	
Often to frequently	8 (34.8)	9 (25.0)		6 (33.3)	8 (27.6)	
All the time	1 (4.3)	2 (5.6)		1 (5.6)	1 (3.4)	
Irregular vaginal bleeding			0.744			0.782
Never or rarely	7 (30.4)	13 (35.1)		8 (44.4)	10 (34.5)	
Often to frequently	9 (39.1)	16 (43.2)		6 (33.3)	12 (41.4)	
All the time	7 (30.4)	8 (21.6)		4 (22.2)	7 (24.1)	
Discomfort with irregular vaginal bleeding (from 0 to 10), mean ± sd	5.2 ± 3.6	4.5 ± 3.4	0.455	3.2 ± 3.9	4.8 ± 3.7	0.182
Overall tolerance (from 0 to 10), mean ± sd	6.7 ± 3.2	7.5 ± 2.4	0.300	7.8 ± 2.5	6.8 ± 2.6	0.191
Overall satisfaction (from 0 to 10), mean ± sd	7.2 ± 3.0	6.8 ± 3.0	0.641	**8.5 ± 1.7**	**6.6 ± 2.9**	**0.012**
Desire to continue the contraceptive method	20 (87.0)	29 (80.6)	0.523	16 (80.0)	21 (72.4)	0.544

*ENG-Implant* Etonogestrel-releasing contraceptive implant; *DSG + ENG-Implant* Daily oral 75 µg of desogestrel (DSG) for 3 months prior to the insertion of ENG-Implant; *SD*  Standard Deviation; *n*  number; *3 M* assessment at 3 months since the beginning of the study; *12 M*  assessment at 12 months since ENG-Implant insertion

Factors associated with contraceptive discontinuation at 3 and 12- months are represented in Table 2**.** Statistically significant side effects associated with ENG-IMPLANT discontinuation at 3 months were mood swings, reduced sex drive and irregular vaginal bleeding. Reduced sex drive was also found to be a reason for DSG discontinuation at 3 months. Women who experienced mastodynia and irregular bleeding were also more likely to discontinue the Implant at 12 months in the DSG + ENG-IMPLANT group. There was a statistically significant improvement over time for both groups concerning the reporting of headache, acne and abdominal pain.

**Table 2:** Discontinuation rates of contraceptive method at 3 and 12- months according to side effects

Variable	ENG-Implant				DSG + ENG-Implant			
	3 M		12 M		3 M		15 M	
	n (%)	P value	n (%)	P value	n (%)	P value	n (%)	P value
Total	3		4		7		8	
Weight gain		**0.025**		0.787		0.574		0.658
No	0		1 (10.0)		6 (21.4)		4 (30.8)	
Yes	3 (33.3)		1 (14.3)		1 (12.5)		3 (23.1)	
Headache		0.955		0.502		0.868		0.502
Never or rarely	2 (13.3)		2 (13.3)		5 (29.8)		2 (13.3)	
Often to frequently	1 (12.5)		0		2 (18.2)		0	
Acne		0.168		0.502		0.124		0.128
Never or rarely	0		2 (13.3)		2 (9.1)		4 (18.2)	
Often to frequently	1 (14.3)		0		4 (33.3)		3 (60.0)	
All the time	1 (33.3)		_		1 (50.0)		1 (50.0)	
Abdominal pain/bloating		0.484		0.502		0.273		0.299
Never or rarely	1 (8.3)		2 (13.3)		5 (23.8)		5 (22.7)	
Often to frequently	2 (18.2)		0		1 (7.7)		3 (42.9)	
Mastodynia		0.567		0.596		0.701		**0.009**
Never or rarely	3 (14.3)		2 (12.5)		6 (20.7)		3 (14.3)	
Often to frequently	0		0		1 (14.3)		5 (62.5)	
Vaginal infection		0.263		0.799		0.766		0.603
Never or rarely	2 (10.0)		2 (13.3)		6 (18.8)		6 (24.0)	
Often to frequently	1 (33.3)		0		1 (25.0)		1 (50.0)	
Mood Swings		**0.012**		0.289		0.386		0.128
Never or rarely	2 (20.0)		2 (15.7)		3 (14.3)		2 (12.5)	
Often to frequently	0		0		4 (30.8)		4 (44.4)	
All the time	1 (100.0)		_		0		2 (50.0	
Reduced sex drive		**0.029**		0.836		**0.012**		0.657
Never or rarely	1 (7.1)		1 (9.1)		3 (12.5)		5 (25.0)	
Often to frequently	1 (12.5)		1 (16.7)		2 (22.2)		3 (37.5)	
All the time	1 (100.0)		0		2 (100.0)		0	
Irregular vaginal bleeding		**0.019**		0.461		0.862		**0.010**
Never or rarely	0		1 (12.5)		2 (15.4)		2 (20.0)	
Often to frequently	0		0		3 (20.0)		1 (8.3)	
Constant	3 (42.9)		1 (25.0)		2 (25.0)		5 (71.4)	

*ENG-Implant*  Etonogestrel-releasing contraceptive implant; *DSG + ENG-Implant*  Daily oral 75 µg of desogestrel (DSG) for 3 months prior to the insertion of ENG-implant; *n*  number; *3 M* assessment at 3 months; *12 M*  assessment at 12 months.

Additional file 2: Table S2 reports patients’ satisfaction, at 3 and 12- months assessment, for the DSG + ENG-IMPLANT group in terms of overall tolerance and satisfaction according to various categories with scores ranging from poor and medium (score ≤ 5) to good and excellent (score > 5). According to this calculation, women who tolerated well the DSG pill at 3 months (score > 5) did not seem to better tolerate the implant at 12 months, as the proportion of women reporting good to excellent tolerance at 12 months was lower compared to the ENG-IMPLANT group (58.6% vs. 88.9% in DSG + ENG-IMPLANT and ENG-IMPLANT group, respectively, p = 0.027) and the continuation rate at 12 months seemed even lower, though not statistically significant (72.4% vs. 80.0%, p = 0.544).

### Discussion

#### Findings, their interpretation and comparison to other studies

There have been numerous studies evaluating ENG-IMPLANT side effects but no trial, to our knowledge, who evaluate whether the practice of 3 months oral intake of DSG prior to ENG-IMPLANT insertion improves its continuation rate at-1-year.

There was no statistically significant difference regarding overall satisfaction or method continuation when using DSG for 3 months prior to ENG-IMPLANT insertion. Women who tolerated well the DSG pill at 3 months (score > 5) did not seem to better tolerate the implant at 12 months, since the proportion of women reporting good to excellent tolerance at 12 months was lower compared to the ENG-IMPLANT group alone. This might suggest that using DSG in order to predict or improve the continuation rate at one year of ENG-IMPLANT is not a promising strategy.

Analysis of side effects reported in both groups did not show a difference in average tolerance between the two groups at 3 and 12 months. However, women reported a higher level of satisfaction in the ENG-IMPLANT group than the DSG + ENG-IMPLANT group at 12 months (8.5 vs. 6.6, p = 0.012). The incidence of side effects with ENG-IMPLANT at 3 and 12 months are congruent with previous publications [1, 8, 9]. Of notice, in both groups, with time, headache, acne and abdominal pain improved, similar to other reporting [1]. These results had no effect on the desire to continue with the implant after 12 months, which was similar between groups (80.0% vs 72.4%, ENG-IMPLANT and DSG + ENG-IMPLANT groups, respectively). The continuation rates with ENG-IMPLANT in our cohort are similar to those reported in the literature (70–75%) [5, 8], comparable to intrauterine methods adherence (73–91%) [9, 10], however, higher than with oral contraceptive use (32–68%) [4, 8].

The higher level of satisfaction at 12 months in the ENG-IMPLANT group compared with the DSG + ENG-IMPLANT group is probably related to the fact that the study was offered only to patients interested in a long-acting reversible contraception willing to accept the possibility of a randomisation to an initial 3 months period of pill intake. Patients’ self-determination when choosing a contraceptive method has shown to ultimately result in greater satisfaction and higher continuation rates [11], which could explain why women who wanted the implant from the outset and received it, were more satisfied.

### Conclusion

In conclusion, although our small sample size, it seems that the use of DSG prior to ENG-IMPLANT insertion does not help in predicting the continuation rate of the implant at one year.

## Limitations

The main limitation of our study is the small number of patients included and the high rate of loss at follow-up. Therefore, no definitive conclusions can be drawn to enforce change in attitude.

## Supplementary Information

**Additional file 1: Table S1.** Baseline sociodemographic and reproductive health characteristics of study participants

**Additional file 2: Table S2.** Satisfaction results at 3 and 12- month assessment on the DSG + ENG-Implant group

## Data Availability

According to Institutional Policies the database will be available upon request.

## Abbreviations

DSG: Daily oral 75 µg of Desogestrel
ENG-IMPLANT: Etonogestrel-releasing contraceptive implant
