# Supplementary material for: Can a 3 months treatment with oral Desogestrel prior to insertion of the etonogestrel-releasing contraceptive implant improve continuation rate at 1 year? A randomized trial
Source: BMC Res Notes. 2023 Mar 13;16:35. doi: 10.1186/s13104-023-06304-3 (PMC10010063; doi:10.1186/s13104-023-06304-3)
Supplement: Supplementary file 1 — Additional file 1: Table S1. Baseline sociodemographic and reproductive health characteristics of study participants [file 13104_2023_6304_MOESM1_ESM.pdf]

Table S1. Baseline sociodemographic and reproductive health characteristics of study participants

Variable	ENG-Implant group n (%)	DSG + ENG-Implant group n (%)	Total n (%)	P value
Participants recruited	23 (38.3)	37 (61.7)	60	
Age in years, median (IQR)	25 (20-30)	24.5 (21-31)	25 (20-31)	0.932
Marital status				0.380
With partner	20 (87.0)	32 (86.5)	52 (86.7)	
Single	3 (13.0)	5 (13.5)	8 (13.3)	
Employment status				0.618
Employed with tertiary education	2 (9.5)	7 (19.4)	9 (15.8)	
Employed with obligatory education	6 (28.6)	12 (33.3)	18 (31.6)	
Student	11 (52.4)	14 (36.1)	24 (42.1)	
Unemployed/without insurance	2 (9.5)	4 (11.1)	6 (10.5)	
BMI (mean±sd)	23.6±4.8	22.8±3.0	23.1±3.8	0.477
Parity				0.936
Nulliparous	17 (73.9)	27 (73.0)	44 (73.3)	
1 to 4	6 (26.1)	10 (27.0)	16 (26.7)	
Previous contraception				0.804
No	9 (39.1)	11 (31.4)	20 (34.5)	
Combined Hormonal Pill	3 (13.1)	5 (14.3)	8 (13.8)	
Condom	7 (30.4)	13 (37.1)	20 (34.5)	
Progestative Pill	1 (4.3)	3 (8.6)	4 (6.9)	
Copper IUD	3 (13.1)	2 (5.7)	5 (8.6)	
Injection	0	1 (2.9)	1 (1.7)	
Tobacco smoking				0.799
Yes	12 (52.2)	20 (55.6)	32 (54.2)	
No	11 (47.8)	16 (44.4)	27 (45.8)	

Abbreviations: ENG-Implant = Etonogestrel-releasing contraceptive implant; DSG+ENG-Implant = Daily oral 75µg of desogestrel (DSG) for 3 months prior to the insertion of ENG-Implant.
