# Supplementary material for: Can a 3 months treatment with oral Desogestrel prior to insertion of the etonogestrel-releasing contraceptive implant improve continuation rate at 1 year? A randomized trial
Source: BMC Res Notes. 2023 Mar 13;16:35. doi: 10.1186/s13104-023-06304-3 (PMC10010063; doi:10.1186/s13104-023-06304-3)
Supplement: Supplementary file 2 — Additional file 2: Table S2. Satisfaction results at 3 and 12- month assessment on the DSG + ENG-Implant group [file 13104_2023_6304_MOESM2_ESM.pdf]

Table S2. Satisfaction results at 3 and 12- month assessment on the DSG + ENG-Implant group

**Variable**	**3M**	**12M**	**P value**
Overall tolerance (from 0 to 10)			0.027
≤5	6 (16.7)	12 (41.4)	
>5	30 (83.3)	17 (58.6)	
Overall satisfaction (from 0 to 10)			0.504
≤5	12 (33.3)	12 (41.4)	
>5	24 (66.7)	17 (58.6)	
Desire to continue the contraceptive method			0.814
Yes	27 (75.0)	21 (72.4)	
No	9 (25.0)	8 (27.6)	

Overall tolerance: poor and medium (score ≤5), good and excellent (score >5)
Overall satisfaction: poor and medium (score ≤5), good and excellent (score >5)
